# Optimizing nutritionally adequate food basket using linear programming in Niayes Households, Senegal

**DOI:** 10.1371/journal.pone.0343156

**Published:** 2026-02-18

**Authors:** Cheikhou Tidiane Willane, Papa Mamadou Dit Doudou Sylla, Mbeugué Thiam, Babacar Mbaye Ndiaye, Laure Tall, Nicole Idohou-Dossou, Adama Diouf

**Affiliations:** 1 Laboratoire de Recherche en Nutrition et Alimentation Humaine, Département de Biologie Animale, Faculté des Sciences et Techniques, Université Cheikh Anta Diop (UCAD), Dakar, Sénégal; 2 Laboratoire des Sciences Biologiques, Agronomiques, Alimentaires et de Modélisation des Systèmes Complexes, UFR des Sciences Agronomiques de l’Aquaculture et des Technologies Alimentaires, Université Gaston Berger de Saint-Louis, Saint-Louis, Sénégal; 3 Laboratoire de Mathématiques de la Décision et de l’Analyse Numérique, Faculté des Sciences Économique et de Gestion, Université Cheikh Anta Diop (UCAD), Dakar, Sénégal; 4 Initiative Prospective Agricole et Rurale, Dakar, Sénégal; Universidad de Guadalajara, MEXICO

## Abstract

In Senegal, particularly in rural areas, diets are often monotonous and primarily based on cereals, with limited intake of animal proteins, vegetables, fruits, and dairy products. The COVID-19 pandemic has exacerbated this situation by increasing the prices of staple foods, further reducing the quality of household diets. This study aimed to develop a nutritionally adequate and food basket adapted to households in the Niayes area, located along the northern maritime fringe of Senegal. A cross-sectional survey was conducted among 90 households to assess dietary diversity of the households and the adequacy of nutrient intake by the daily diet, among vulnerable groups such as women of reproductive age, children under five years old, and elderly individuals (60 years and above). Household dietary diversity was assessed using a 24-hour recall, with high dietary diversity defined as the consumption of at least eight out of twelve food groups. Foods commonly consumed by households were listed and their prices were collected from local food retailers identified through household purchasing practices. Linear programming (LP) was used to optimize a low-cost food basket covering daily energy and micronutrient requirements. Around 44.4% of households exhibited high dietary diversity. The coverage of daily requirements for certain micronutrients, including calcium, folate, and vitamin D, among vulnerable groups was less than 50%. The optimized food basket, costing 6,917 XOF (~11.48 USD) per day, contained nine food groups and successfully covered the daily energy, protein, and micronutrient requirements of a reference household of 13 individuals. These results highlight the potential of linear programming as a decision-support tool for designing nutritionally adequate food baskets adapted to local contexts.

## Introduction

In developing countries, monotonous diets primarily composed of starchy staples and lacking in animal-source foods, fruits, and vegetables remain a major concern [1]. Furthermore, the affordability and availability of nutritionally poor foods contribute to inadequate diets, particularly among low-income populations [2].

In Senegal, 70.3% of households have a moderate dietary diversity (4–5 food groups), while 19% have a poor food consumption score defined by diets dominated by cereals, oil, sugar and sweet products, and only occasional consumption of vegetables, which provide mainly energy but limited essential proteins and micronutrients [3,4]. Overall, 16.3% of rural households have an inadequate food consumption score (poor or borderline), compared to 9.7% for households residing in urban areas [5].

These trends are linked to high prevalence of malnutrition, especially among children and women of reproductive age. According to the National Food Security Council in Senegal [4], the prevalence of global acute malnutrition in Senegal is 18.8% among women aged 15–49 years and 13.4% among children aged 6–59 months. Around 18.3% of children under 5 years are stunted and 16.7% are underweight.

This situation has been further exacerbated by the COVID-19 pandemic, which has significantly impacted household economies. According to the World Bank [6], food price inflation rose sharply during the pandemic, leading vulnerable populations to reduce both the quantity and quality of their food intake.

In Sub-Saharan Africa, restrictive public health measures disrupted food supply chains, resulting in food shortages and higher prices for nutritious items [7]. In Senegal, the closure of weekly markets led to a dramatic increase in staple food prices due to reduced availability. To mitigate these effects, government agencies and Non-governmental organization (NGO) have distributed food kits to low-income households. Nearly one million households benefited from these kits, which typically included oil, rice, pasta, and sugar [8]. The Food and Agriculture Organization of the United Nations (FAO) also distributed more diversified kits containing cereals, fish products, eggs, and vegetables. Although most of these interventions lacked a clear nutritional rationale and did not consider local dietary preferences, food kits were nonetheless distributed during emergency situations such as the COVID-19 pandemic, highlighting the need for context-specific and nutritionally informed strategies. [9].

Linear programming (LP) offers a promising tool for designing nutritionally adequate and cost-effective food baskets, particularly in emergency contexts. This method has been used in several countries, such as Honduras [10], Ethiopia [11] to develop affordable diets that cover nutritional requirements. This study is part of the project Responding to COVID-19 through Social Protection and Strengthening Local Food Systems: The Case of the Niayes in Senegal (COPSA), which was implemented by the Initiative Prospective Agricole et Rurale (IPAR) and the International Development Research Centre (IDRC). This study, aims to assess the dietary diversity of households in the Niayes area and the coverage of daily nutritional requirement of vulnerable groups (children, women and the elderly) by the daily diet of households. We also sought to apply linear programming (LP) to design a nutritionally adequate food basket at minimum cost for households in the Niayes region of Senegal.

## Materials and methods

### Subjects and study design

This cross sectional study was conducted in households located in the Niayes area, a strip of land stretching from Dakar to Saint Louis over a length of 180 km and a width varying from 5 to 30 km [12]. Niayes area is located in the north-west of Senegal and is characterized by depressions and dunes resting on a shallow water table. It covers four administrative subdivisions: Dakar, Thies, Louga and Saint Louis [13]. It has biophysical characteristics favourable to intensive agro-pastoral activities and provides the bulk of Senegal’s vegetable production, over 80% [14,15] and is the first fishing region of Senegal.

The study was conducted as part of a study on response to covid-19 through social protection and strengthening of local food systems: the case of the niayes in Senegal (COPSA) [16]. The survey was conducted in 90 households randomly selected from the sub-sample of the COPSA study. Data collection was carried out between February and March 2022. Household dietary diversity was collected before randomly selecting one person from vulnerable group (child 6–23 months, 24–59 months, pregnant woman, breastfeeding woman, man and woman 60 years of age or beyond) and weighing all the foods consumed from morning to evening in order to assess the nutrient intake from the household’s daily diet. Consent was obtained from all household representatives prior to data collection. Participants were given a consent letter, which they read and signed after receiving an explanation of the study objectives, procedures, and confidentiality measures. In the case of minors (less than 18 years), their parents or legal guardian has signed the consent form.

## Data collection

### Dietary assessment

Dietary and nutrient intakes were measured by weight food record method. The dietary diversity (DD) of households was assessed using 24h dietary recall questionnaire. These data were collected by eight interviewers and supervised by two nutritionists. The interviewers were trained for five consecutive days, followed by a pretest on questionnaire administration and food weighing.

### Weight food record

All foods and beverages consumed during a whole day (24 hours, from waking to bedtime) by an individual from vulnerable group (child 6–23 months, 24–59 months, pregnant woman, breastfeeding woman, man and woman 60 years of age or beyond) randomly selected in the household was quantify. Weighing was carried out using dietary scales with a maximum capacity of 5 kg and an accuracy of 1g (Gram, Barcelona, Spain; Soenhle, Nassau, Allemagne; Tefal, Ecully, France). Simple foods were weighed before consumption. For mixed dish, the composition was noted and each ingredient weighed before and after cooking. The total amount of each mixed dish after cooking was recorded. The quantity consumed by individual during a meal was obtained by multiplying the weight of a standard handful/spoon with all the ingredients usually consumed by the total number of handfuls/spoons ingested [17].

The individual’s dietary intakes were estimated on a raw weight basis by multiplying the multiplication factor and the quantity before cooking each ingredient of the dish. Multiplication factor was obtained by the ratio between the quantity of food consumed by the household and the quantity of food consumed by an individual.

### Nutrient intakes by household diet and nutritional requirements coverage

Daily nutrient intakes (energy, protein, calcium, iron, zinc, vitamin A, vitamin D and folate) was determined using the FAO/INFOODS West African food composition table [18]. These nutrients were selected due to their critical role in growth, immune function, and the high prevalence of deficiencies among vulnerable groups in Senegal, including children, pregnant and breastfeeding women, and the elderly [19,20]. Nutrient coverage for vulnerable groups (children, pregnant and breastfeeding women, elderly) among households was calculated based on Dietary Reference Intakes (DRI) [21,22]. Estimated Energy Requirements (EER) for each vulnerable group was based on Institute of Medicine (IOM) formulas that take into account of age, gender, weight, height and physical activity level [22]. For iron and zinc, coverage levels were calculated based on their bioavailability (10% for iron and 30% for zinc) in household diets [21].

### Household dietary diversity measurement

The 24-hour recall was conducted during an interview in which the interviewer asked the woman who prepared the household meals to recall and describe all the foods and beverages consumed by the household during the 24 hours preceding the survey. All foods consumed by the household were classified into 12 foods groups: cereals; white roots and tubers; legumes, nuts and seeds; vegetables; fruits; milk and dairy products; meat, poultry and offal; oils and fats; fish and seafood; sweets; eggs; and spices, condiments, and beverages. Household dietary diversity was assessed based on the proportion of households consuming at least eight of the twelve predefined food groups. Dietary diversity was classified as moderate when households consumed between six and eight food groups, and as low when five or less out of the 12 groups were consumed [23,24].

### Foods, prices and categorization

Foods available in the area were listed from different food retailers (markets, neighbourhood shops, etc.). In each locality, retail outelets where households purchasing were targeted, and questionnaires were addressed to shopkeepers/vendors to list raw/uncooked foods and ready-to-eat products and their prices. Only raw or minimally processed foods (e.g: peanut powder, peanut paste) often consumed were listed. Between 1–3 different prices were collected for each food. For all items the price per kilogram or liter was collected (S1 Table). If prices were provided per piece (e.g., for lemon, melon, fish), their reference weights were used to calculate the price per kilogram. The average price was used for food basket optimization and conversions were calculated to be presented in XOF/gram. All the foods listed were classified into the aforementioned 12 food groups defined for calculating the household dietary diversity [23].

### Nutritional composition

Data of the food nutritional composition were obtained from the West African food composition table [18] and Nutrisurvey/ENA software (https://www.nutrisurvey.de/ena/ena.html). Information on the non-edible portions of foods was taken from the same databases.

### Reference household

Size of Reference Household was defined based on the average household size in Niayes area (n = 13). The Food Basket (FB) was calculated for a reference household (Table 1) of 13 individuals composed of: 1 child aged 6–23 months, 24–59 months, 5–9 years, 1 boy 10–19 years, 2 girls aged 10–19 years, 1 non-pregnant non-breastfeeding woman, 1 pregnant woman, 1 breastfeeding woman, 1 woman aged 60 years and more, 2 men 19–59 years and 1 man aged 60 years old or more [25].

**Table 1:** Reference household.

Age groups	Household composition
Child 6–23 months	1
Child 24–59 months	1
Child 5–9 years	1
Boy 10–19 years	1
Girls 10–19 years	2
Non-Pregnant Non- Breastfeeding woman (NPNBW)	1
Pregnant woman	1
Breastfeeding woman	1
Woman 60 years and more	1
Men 19–59 years	2
Man 60 years and more	1

For adolescents and adults, the optimized nutrient requirements and food baskets reflect the actual composition of the reference household. Specifically, the food baskets and nutrient requirements presented for girls aged 10–19 years correspond to two individuals, as the reference household includes two adolescent girls. Similarly, food baskets and nutrient requirements for adult men aged 19–59 years represent two individuals. Therefore, the reported food quantities and costs reflect the combined amounts for two persons.

A food basket was optimised for each household member and the individuals optimized FBs were combined to give the total daily FB for the household.

### Energy requirements and recommended nutrient intakes

Estimated Energy Requirements, DRI and tolerable upper limits for some nutrients were defined for each individual of the reference household. The EERs were calculated for each member used Institute Of Medicine [22] formulas based on age, sex, weight, height and Physical Activity Level (PAL). PAL for each age group was obtained from the literature review [26–29]. DRIs had been determined based IOM and FAO recommendations [21,22]. **Table 2** provides information on EERs and DRIs used as constraints during LP.

**Table 2:** Energy and Dietary Reference Intakes applied during linear programming.

	Children			Adolescents (10–19 ye)		Women				Men	
Energy/Nutrients	6-23 mo	24-59 mo	6-9 ye	Boy	Girls	NPNB	Pregnant	Breastfeeding	60 ye and more	19-59 ye	60 ye and more
Energy kcal/d	696	1 491	1631	2 448	3 934	2 204	2 656	2 604	1 713	5 844	2 294
Protein (g/d)	11	13	19	52	92	46	71	71	46	112	56
Fat (g/d)	37	45	53	78	114	62	67	75	51	162	57
Carbohydrate (g/d)	130	130	130	130	260	130	175	210	130	260	130
Fiber (g/d)	<19	19	25	31	52	25	28	29	21	76	30
Calcium (mg/d)	400	500	700	1 300	2 600	1 000	1 200	1 000	1 300	2 000	1 300
Iron (mg/d)	7,55	6,3	8,9	18,8	65,4	29,4	27	15	11,3	27,4	13,7
Magnesium (mg/d)	60	76	100	230	440	220	220	270	190	520	224
Phosphorus (mg/d)	275	460	500	1 250	2 500	700	883	883	700	1 400	700
Potassium (mg/d)	1 430	2 000	2 300	2 750	4 600	2 600	2 700	2 600	2 600	6 800	3 400
Sodium (mg/d)	800	900	1 000	1 350	2 700	1 500	1 500	1 500	1 500	3 000	1 500
Zinc (mg/d)	4,1	4,8	5,6	8,6	14,4	4,9	10	9,5	4,9	14	7
Vit A (µgRE/d)	185	200	250	330-399	660-800	270	370	450	300	600	300
Vit D (µg/d)	5	5	5	5	10	8	5	5	15	16	15
Vit E (mg/d)	5	6	7	13	26	15	15	15	15	30	15
Folate (µg/d)	80	200	300	400	800	400	600	500	400	800	400
Vit B12 (µg/d)	0,7	0,9	1,8	2,4	4,8	2,4	2,6	2,8	2,4	4,8	2,4
Vit C (mg/d)	30	30	35	40	80	45	55	70	45	90	45

*NPNB: Non-Pregnant Non-Breastfeeding; Vit: Vitamin; d: day*; mo: months; ye: years

### Linear programming and food basket optimization

Linear programming is a mathematical method for optimization (minimization or maximization) of a given linear goal function (objective function) submitted to a series of constraints on a list of decision variables [30]. The model description is based on three major elements: objective function, defined as a linear combination of decision variables that is either minimized or maximized; decision variables, which are the quantities of food to be included in the optimized FB; and a set of constraints (criteria to be respected) [31]. Applied to nutrition, it can be used to identify food combinations that comply with a range of nutritional recommendations by providing rations that are both acceptable and realistic [32].

For this study, a general linear programming model was proposed with the aim to develop an optimal food basket to cover all the household members nutritional requirements at a relatively low cost that can be used in emergency situations. The objective function was to minimize the total diet cost while satisfying a set of predefined nutritional constraints, using a linear programming model solved with the simplex algorithm. Linear optimization was done with the COIN-OR CBC optimization engine algorithm, which is part of the open source add-in OpenSolver. This open source OpenSolver (v. 15.0) is an add-in for Microsoft Excel, designed to solve linear and integer programs in Excel [33].

### Decision variables

The foods listed and included in the model were considered to be the decision variables.

x_j_ = Quantities of food j to consume per day (in grams)

aj = Cost of each food

**1. Objective function**

The objective function used aimed to minimize the cost of the FB and was expressed as follows:


$$\text{Min y}=\sum_{j=\text{1}}^{n}{\text{a}}_{\text{j}}{\text{x}}_{\text{j}}$$

a_j_: unit price of food j

n = Number of foods included in the model

**2. Constraints**

The constraints used in this model were divided into 4 groups which are the following:

1. Energy requirements

$$\sum {\text{x}}_{\text{j}}\times {\text{E}}_{\text{i}}\geq {\text{B}}_{\text{0}}$$

E_i_: amount of energy provided by 1 g of food

B_0_: total energy requirement (kcal) per day

2. Recommended nutrient intake

$$\sum_{j}^{m}{\text{z}}_{\text{ij}}{\text{x}}_{\text{j}}\geq {\text{k}}_{\text{i}}\;\forall_{j}=\text{1},\;\ldots ,\text{ m}$$

k_i_: recommended daily allowance of nutrient i

z_ij_: quantity of nutrient i per gram of food j

m: Number of nutrients considered in the model

3. Tolerable upper limits not to be exceeded for some nutrients (sodium, vitamin A) had been used as constraints. These describe the maximum quantity of different nutrients that can be consumed on a daily basis, so as to avoid negative secondary effects.

$$\sum_{j}^{m}{\text{z}}_{\text{ij}}{\text{x}}_{\text{j}}\leq {\text{w}}_{\text{i}}\;\forall_{j}=\text{1},\;\ldots ,\text{ m}$$

w_i_: tolerable upper limit of nutrient i

4. The maximum quantity of each food not to be exceeded per day and minimum quantity [23] for certain foods to be included in the optimization FB.

$${\text{t}}_{\text{j}}\leq {\text{x}}_{\text{j}}\leq {\text{T}}_{\text{j}}\;\forall_{\text{j}}=\text{1},\ldots ,\text{ n}$$

tj = Minimum quantity of food j to be included in the optimization FB

T_j_= Maximum quantity of food j to consume during the day

## Results

### Daily nutrient intakes and coverage

**Fig 1:**
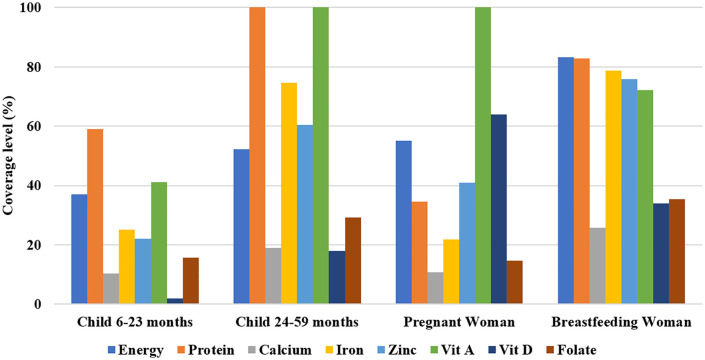
Level of coverage of daily nutrient requirements for children, pregnant and breastfeeding woman.

**Fig 2:**
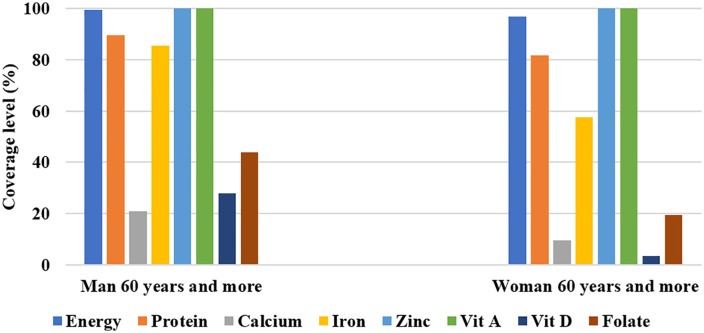
Level of coverage of daily nutrient requirements for man over 60 years and woman over 60 years.

Daily energy and nutrient intake coverage were calculated from the household dietary survey and refers to the vulnerable groups included in the study. Median energy intakes were low for children aged 6–23 months (257.4 kcal/d [79.7; 297.4]), 24–59 months (778.9 kcal/d [590.5; 930.2]) and pregnant women (1463 kcal/d [1330; 1597]) (**Table 3**). The diet covered 37%, 52% and 55% of their energy requirements, respectively (Fig 1). However, among breastfeeding women, women aged 60 and more and men aged 60 and more, daily energy and protein requirements were covered at over 80% (Fig 1 and 2). Median protein intake for pregnant women and children aged 6–23 months was 24.6 g/d [23.3; 25.8]) and (6.5 g/d [3.0; 8.5], respectively. Protein requirement were covered at 40% and 59.1% for their two groups. Coverage level of iron requirement by household diet was low among children aged 6–23 months and pregnant women (<25%). While, for zinc, calcium, vitamin D and folate, median intakes and coverage level were low for all vulnerable groups. For children aged 24–59 months, daily vitamin A requirements were fully covered by the diet, and those for calcium (19%), vitamin D (18%) and folates (29%) are only slightly covered. For pregnant women, only the daily requirement of vitamin A was 100% covered by the diet, while the daily requirements for calcium (11%), and folates (15%) were poorly covered. For breastfeeding women, no micronutrient requirement was fully covered by the diet, calcium (26%), vitamin D (34%) and folate (35%) were the least covered. For the elderly men 60 years and more and women 60 years and more, vitamin D (28% and 3.3%), calcium (20.9% and 9.6%) and folate (44% and 19.4%) requirements were the least covered.

**Table 3:** Daily nutritional intake of the diet of the Niayes area households assessed among children, women and the elderly.

	Children		Women		60 years and more	
**Energy/Nutrients**	**6-23 months**	**24-59 months**	**Pregnant**	**Breastfeeding**	**Man**	**Woman**
Energy (kcal)	257.4 [79.7; 297.4]	778.9 [590.5; 930.2]	1 463 [1 330; 1597]	2 170 [1 619; 2462]	2 282 [1 414; 2397]	1 658 [1 360; 2051]
Protein (g/d)	6.5 [3.0; 8.5]	25.9 [15.6; 28.0]	24.6 [23.3; 25.8]	58.8 [36.6; 71.1]	50.2 [34.5; 66.5]	37.6 [27.5; 43.8]
Calcium (mg/d)	41.2 [23.9; 57.2]	95.2 [64.4; 102.4]	130.1 [119.1; 141.1]	256.9 [231.3; 266.4]	272.1 [180.6; 442.1]	125.3 [194.1. 157.9]
Iron (mg/d)	1.9 [0.5; 3.5]	4.7 [2.8; 5.3]	5.9 [5.6; 6.3]	11.9 [9.6; 14.8]	11.7 [11.5. 16.6]	6.5 [5.1; 8.0]
Zinc (mg/d)	0.9 [0.4; 1.4]	2.9 [2.1; 3.4]	4.1 [4.0; 4.1]	7.2 [5.9; 9.4]	7.9 [6.1; 9.4]	5 [4.6; 6.7]
Vitamin A (µgRE/d)	76.2 [57.8; 107.4]	478.5 [171.8; 576.3]	1 303 [1 250; 1357]	1 493 [1 306; 1572]	1 155 [763.7; 1754.5]	1 011.7 [956.5; 1 383.8]
Vitamin D (µg/d)	0.1 [0.02; 0.3]	0.9 [0.4; 1.1]	3.2 [2.0; 4.5]	1.7 [0.7; 3.3]	4.2 [1.8; 6.1]	0.5 [0.2; 1.4]
Folate (µg/d)	12.5 [6.6; 29.0]	58.5 [42.2; 93.4]	87.4 [77.6; 97.2]	176.5 [142.2; 282.1]	176.2 [148.5; 215.7]	77.5 [66.0; 94.9]

### Household dietary diversity

The mean dietary diversity score of the household of Niayes area was 8.5 ± 1.1 and 55.6% of them had consumed at least 8 food group. However, 44.4% had high dietary diversity (Table 4).

**Table 4:** Household dietary diversity.

	M ± Sd or %	Effective (n = 90)
Household Food Diversity Score	8.5 ± 1.1	90
Moderate food diversity (*6–8 food groups*)	55.6	50
High dietary diversity (*> 8 food groups*)	44.4	40

## Individuals foods baskets

Individual FB were developed using linear programming optimization to meet all nutritional requirements of each household member at minimum cost**.** Table 5 showed the composition of the FBs for each household members. The FB for children aged 6-23 months (10 items) was the least expensive (159 XOF per day), followed by that for children aged 24-59 months (200 XOF per day), which also contained the fewest foods (7 items). The FB for pregnant woman was the most expensive, costing 815 XOF per day and containing the most foods (24 items). All the FBs contain rice and only the child 6-23 months and child 24-59 months FBs didn’t contain sardine fish. The results also showed that only the foods baskets of girls 10-19 years didn’t contain fruits. No foods from the vegetable group were present in the food basket of a man aged 60 and more. However, for woman 60 and more, only 2 foods from this group (carrot and cabbage) were present in the food basket.

**Table 5:** Foods baskets for each household member.

		Children			Adolescents (10–19 years)		Women				Men	
Foods groups	Foods	6 to 23 months	24–59 months	5 to 9 years	Girls	Boy	NPNB	Pregnant	Breastfeeding	60 years and more	19 to 59 years	60 years and more
Cereals	Bread	56.4										
	Rice	6.6	141.8	48.2	24.2	46.9	117.5	113.6	86.1	133.2	62.4	65.8
	Millet			24.5	22.6							
	Maize	54.3			303.4						75.2	
White roots and tubers	Cassava							34.1	88.2			
	Turnip							88.5	88.5			
Pulses, nuts and seeds	Peanut Powder	29.9	117.9				83.2	83.2	83.2		330.4	134.9
	Peanut Past				152.6	98.2	15	6	41			
	Cowpeas			83.5		14.7		61.5	61.5	49.2	36.6	2.6
	Green bean						22.2	22.2	22.2			
Vegetables	Fresh sorrel leaf		93.3	105.7	246.8	99.7	36.2					
	Carrot	7.6						80.7			13.8	
	Cabbage							287.2	287.2	47.4	96.4	
	Onion								245.8			
	Pepper							13.3				
	Concentrated tomato				238.0		74.9	88.9				
	Dry sorrel calyx	82.5				102.3	7.2	7.2	7.2	102.8		
	Fresh moringa leaf				201.8		37.7	37.7	10.4			
Eggs	Egg			1.6								
Fruit	Tamarind					108.7	19.6	19.6	19.6	105.6	22.8	128.1
	Baobab fruit	12	76.4	35.6		115.7	23.2	13.5	13.5	113.2	234.6	116.3
Oils and fats	Vegetable oil	3.7	1.7	110.7	10.3	5.5	16.3	16.7	16.3	51.3	9.6	5.8
Fish and seafood	Fish ‘diaye’	18.5	18.5			16.5				33.4	0.3	20.1
	Sardine fish			142	427.4	15.4	352.8	352.8	352.8	170.8	454.7	273.4
	Dried fish				12.2			40.6	40.5	104.9	116.8	103
	Smoked fish				37.6	110.4	29.4	29.4	29.4	106.1	239.2	114.6
Spices and condiments	Salt	0.95	5.6	9	19.4	12.4	29.4	12	29.4	5.7	19.2	
	Parsley			101.3	0.8		17	17	17			
	Pepper							5.6	5.7	0.6	8.6	6
	Fermented African locust Bean				3.2	4.4	3.8	8.6	8.6			6.6
	Mint					3.0	3.8	3.8	3.9		2.4	
**Total weight (g)**		**272.5**	**455.2**	**662.1**	**1 700.6**	**753.8**	**889.2**	**1 443.7**	**1 528.6**	**1 024.2**	**1723.0**	**977.2**
**Cost of baskets (XOF)**		**159**	**200**	**311**	**1009**	**472**	**502**	**815**	**777**	**740**	**1199**	**733**

### Household food basket

The household FB contains 31 foods with a total weight of 11461.8g in 9 food groups: cereals, white roots and tubers, legumes, nuts and seeds, vegetables, fruit, eggs, oils/fats, fish and seafood, spices and condiments. The animal proteins in the FB come from eggs, fish and seafood only. This developed FB provides a high consumption of vegetables, fish and baobab fruits, a lower consumption of cereals and a moderate consumption of salt. The total cost of the basket is 6917 XOF (~11.48 USD) per day (Table 6).

**Table 6:** Household food basket.

Foods groups	Foods	Weight (g)
Cereals	Bread	56
	Rice	846
	Millet	47
	Maize	433
White roots and tubers	Cassava	122
	Turnip	177
Legumes nuts and seeds	Groundnut Powder	863
	Groundnut Past	313
	Cowpeas	310
	Green bean	67
Vegetables	Fresh sorrel leaf	582
	Carrot	102
	Cabbage	718
	Onion	246
	Pepper	13
	concentrated tomato	402
	Dry sorrel calyx	309
	Fresh moringa leaf	288
Eggs	Egg	2
Fruits	Tamarind	424
	Baobab fruit	754
Oils and fats	Vegetable oil	250
Fish and seafood	Fish	107
	Sardine fish	2 542
	Dried fish	418
	Smoked fish	696
Spices and condiments	Salt	143
	Parsley	153
	Pepper	27
	Fermented african locust bean	35
	Mint	17
**Total weight**		**11461.8g**
**Total cost of FB 6 917 XOF (~11.48 USD)**		

### Nutrient content of the optimized food basket

The food basket was designed to cover all the daily nutritional requirements of all the household members (13 individuals). The daily energy and protein requirements of all household members were fully covered by the food basket. Vitamins C, A and B12 are the micronutrients covered the most by the food basket, more than doubling the daily requirements of the whole household. Among the minerals, magnesium and sodium were the most abundant, while calcium requirement was the least covered (Table 7**).** However, only the sodium intake from the food basket is very high and exceeds the tolerable upper limits despite the restrictions applied. No others micronutrient intake exceeded these potentially toxic thresholds.

**Table 7:** Nutritional value of the food basket.

	FB Nutritional value	Nutritional requirements	Coverage of requirements levels (%)
**Energy (Kcal/d)**	30 246	27 515	109.9
**Macronutrients (g/d)**			
Protein	2 006.4	589	340.6
Fat	1 072.6	801	133.9
Carbohydrate	2 730.0	1 815	150.4
Fiber	491.4	355	138.4
**Minerals (mg/d)**			
Calcium	13 345.2	13 300	100.3
Iron	343.9	230.8	149.0
Magnesium	8 481.7	2 550	332.6
Phosphorus	28 404.0	10 251	277.1
Potassium	61 087.0	33 780	180.8
Sodium	63 143.9	17 250	366.1
Zinc	176.6	87.8	201.2
**Vitamins**			
Vitamin A (µgRE/d)	26 846.9	4015	668.7
Vitamin D (µg/d)	118.0	94	125.5
Vitamin E (mg/d)	234.5	162	144.8
Folates (µg/d)	6 668.6	4 880	136.7
Vitamin B12 (µg/d)	218.3	28	779.7
Vitamin C (mg/d)	4 080.0	565	722.1

## Discussion

The results of this study showed that household diet was not enough to fulfil the daily requirements of vulnerable individuals. For children aged 6-23 months, daily requirements for energy, iron, zinc, vitamin D and folate are very poorly covered by the daily dietary intake. In contrast, daily vitamin A and protein requirements were 100% covered. In Senegal, these results found in the Niayes region were similar to those found in the Kaffrine region, where similar coverage of required levels of iron, vitamin A and zinc were shown for children aged 6 to 23 months [34]. Similar results were also found in Madagascar for children aged 1-59 months, where iron and zinc coverage levels do not exceed 70% and 40% respectively, while protein and vitamin A coverage levels are 160% and 120% [35]. The level of nutritional coverage for energy, iron and zinc found in our study could be explained by the household diet, based essentially on cereals and vegetables.

Among pregnant and breastfeeding women, our study showed that daily requirements for iron, calcium, folate and vitamin D are the lowest. Similar results were reported in Tunisia, where daily calcium, iron and zinc intakes in the diet of breastfeeding women were insufficient to cover daily requirements for these nutrients [36]. Comparable results were also reported by study conducted in Algeria, which showed that daily vitamin D and iron intakes failed to cover daily requirements for these nutrients of pregnant women [37].

In this Niayes area, daily energy, iron and zinc requirements are high among the elderly. However, the daily household diet provides lower calcium, vitamin D and folate requirements. This low intake’s level of these nutrients could be the result of limited consumption of fruit and foods of animal origin. It could also be explained by sociocultural factors that characterize Senegalese households.

The average household dietary diversity score was 8.5. Our results are similar to those found in Burkina Faso (average score = 6) and Mali (average score = 6) [38,39]. However, a lower household dietary diversity score (mean score = 4) is found in the Kayes region of Mali and in northern Senegal (mean score = 4.9) [40,41]. The high dietary diversification found among households in this study could be explained by the period during which data collection was conducted. In fact, the Niayes is an area where market gardening and fishing are strongly practiced, a variety of foods are often cultivated in this area, and the data collection was done at the height of the harvest period. Moreover, most of these foods are sold at very low prices.

The results of this study show that the LP methodology can be used to develop an optimized, acceptable and accessible food basket, nutritionally adequate to cover all the nutritional requirement of a household in the Niayes area at a low cost. This FB contained 31 foods: 25.8% legumes; 16.1% spices and condiments; cereals, legumes nuts and seeds, fish and seafood (12.9% each); fruits and white roots and tubers (6.5%) and eggs and oils and fats (3.2% each) and cost 6917 XOF (~11.48 USD) per day.

The price of the basket is slightly higher compared to what researchers found in other African countries such as Ethiopia and Ghana. where the cheapest baskets for a family of five in rural areas and a family of four in rural areas cost 788.7 XOF (~1.31 USD) and 1204.54 XOF (~2.0 USD) per day respectively [11,42]. However. the number of foods in this food basket (31 foods) is higher than those found in these studies (18 and 13 foods respectively). In another study conducted in Denmark and Honduras, the nutritionally adequate food basket developed contained fewer foods than those found in our study [10,43]. Furthermore, the cost of the basket in our study is lower than the daily expenditure of a household of 13 people (~9,113 XOF: USD 16.18) [44].

The slightly higher cost of the basket could be explained by the size of the reference household, containing 13 individuals, which is quite large [13] and by the presence of pregnant and breastfeeding women in the household. These women have high requirements for certain nutrients. Market food prices also vary from one country to another. which could have an impact on the final cost of the basket.

Nevertheless, the low-cost food basket in our study did not contain all 12 food groups defined for household. It is possible that the acceptability of this food basket will be a problem in the long term even if it covers all the daily nutritional requirements. Although, this optimized food basket is nutritionally more adequate, contains more food groups, and costs less than the emergency food aid provided by the Senegalese government during the COVID-19 pandemic, which consisted of only four food groups: cereals, sugar, oil, and pasta [16].

Eggs, fish and fish products are the main sources of animal protein. The presence of fish products can mainly be explained by their relatively low cost in this area, where fishing plays an important role. Meat poultry and offal milk and dairy products and sugar are not in the optimized final basket. The main reason for this could be that including these food groups would considerably increase the cost of the final basket thereby reducing its affordability. Although all animal-based foods are not included in the optimized basket, fish and fish products offer great diversity and are a source of micronutrients (calcium, vitamin D) of animal origin.

This study has some limitations. First, the relatively small sample size does not allow full statistical representativeness of all households in the Niayes area. Therefore, the results should be interpreted as indicative rather than generalizable at the population level. However, the sample was sufficient to support the methodological objective of identifying nutrient gaps and developing a nutritionally adequate and cost-minimized food basket adapted to the local context. The cost of the basket applies only to the purchase of food and does not cover expenses related to transport, cooking equipment and food preparation. The food basket was designed for a reference family in the Niayes area of thirteen people and does not apply to people with special nutritional requirements, such as women who are both pregnant and breastfeeding, people suffering from food intolerances or allergies and sick people. In addition, the availability and prices of collected foods may vary depending on the season, and the absence of national dietary recommendations makes it impossible to assess the cultural acceptability of optimized FB. In addition, the linear programming approach is a normative optimization tool that identifies mathematically optimal food combinations under predefined constraints. It does not account for individual food preferences, cultural acceptability, or intra-household food allocation. Therefore, the optimized food basket should be interpreted as a decision-support tool rather than a prescriptive representation of actual household diets. A limitation of the model is that it may generate food baskets with salt levels above recommended daily intakes, as it prioritizes meeting nutrient requirements, which may not fully reflect safe dietary practices in real-life settings.

## Conclusion

Inadequacy of several nutrients’ intakes were apparent among Niayes households caracterized by low intake of animal based products. Linear programming provided guidelines around the cost of dietary patterns that cover energy and nutrient requirements. Food basket developed for a reference household of 13 members cost 6 917 XOF (~11.48 USD) and contains 31 foods divided into nine [9] food groups.

The use of linear programming to optimize food baskets that meet more nutritional and economic constraints could be further developed in order to improve food diversity and nutrient intake while remaining culturally acceptable. In addition, acceptability, affordability studies of these optimized foods baskets would provide real data and stabilize an economically favorable and nutritionally adequate model that could be used in emergency situations.

## Supporting information

S1 Table Listed foods and their prices/kilogram. (PDF)
