# Supplementary material for: Optimizing nutritionally adequate food basket using linear programming in Niayes Households, Senegal
Source: PLoS One. 2026 Feb 18;21(2):e0343156. doi: 10.1371/journal.pone.0343156 (PMC12915922; doi:10.1371/journal.pone.0343156)
Supplement: S1 Table — (PDF) [file pone.0343156.s001.pdf]

S1 table: Foods and their prices per kilogram/and or liter

Foods	Price 1 (XOF)	Price 2 (XOF)	Price 3 (XOF)	Mean price (XOF)	Unit
**Cereals**					
Fragrant rice	500	500	500	500	Kg
Broken rice	350	325	375	350	Kg
Maroccan couscous	900	800		850	Kg
Wheat	300	300	300	300	Kg
Millet	300	350	400	350	Kg
Maize	225	275	350	283	Kg
Sorghum	400	300		350	Kg
Bread	1000	1000	1000	1000	Kg
**Roots/White tubers**					
Patato	275	400	600	425	Kg
White sweet potato	500	400		450	Kg
Orange-fleshed sweet potato	500	500	500	500	Kg
Cassava	400	450	500	450	Kg
Beetroot	400	500		450	Kg
Turnip	300	450	300	350	Kg
Ginger	1200	1000	1500	1233	Kg
**Legumes, nuts and seeds**					
Cowpea	800	1200	1000	1000	Kg
Green pea	600	700	650	650	Kg
Peanut	750	700		725	Kg
Peanut butter	1200	1000	1100	1100	Kg
Peanut powder	550	600	650	600	Kg
Green Bean	350	300	200	283	Kg
Olive	1300	1300	1300	1300	Kg
White Bean	1800	1500		1650	Kg
**Vegetables**					
Carot	300	400	500	400	Kg
Fresh tomato	250	300	400	317	Kg
Cucumber	600	500		550	Kg
Tomato paste	1200	1600	1400	1400	Kg
Pumpkin	300	400	200	300	Kg
Onion	250	400	275	308	Kg
Eggplant	250	300	200	250	Kg
Parsley	250	350	300	300	Kg
Bitter eggplant	250	300	350	300	Kg
Fresh okra	1500	900		1200	Kg
Fresh sorrel leaves	200	200	200	200	Kg
Cabbage	350	400	500	417	Kg
Bell pepper	250	350	400	333	Kg

Dried sorrel	500	450		475	Kg
Fresh moringa leaves	600	650	475	575	Kg
Green onion	100	200	200	167	Kg
**Fruits**					
Melon	600	700	1000	767	Kg
Papaya	1000	1200		1100	Kg
Watermelon	1000	1500		1250	Kg
Pear	1500	2000	3000	2167	Kg
Grapefruit	500	600		550	Kg
Lemon	400	1200	1500	1033	Kg
Tamarind	600	700	800	700	Kg
Orange	250	400	500	383	Kg
Clementine	900	1000	1200	1033	Kg
Baobab fruit	500	700	600	600	Kg
Banana	700	800	900	800	Kg
Apple	1200	1300	1500	1333	Kg
Grape	3000	4000		3500	Kg
Pineapple	1200	1200	1200	600	Kg
Dates	2000	2000	2000	2000	Kg
Jujubes	800	600		700	Kg
Soump	250	300		275	Kg
Milk and dairy products					
Powdered milk	2500	2400		2450	Kg
Curds	800	750	1000	850	Liter
Cheese	6000	6000	6000	6000	Kg
Cow's milk	750	800	1000	850	Liter
**Meat, poultry, offal**					
Beef	3400	3500	3700	3533	Kg
Mutton	4000	6000	7000	5667	Kg
Liver	4000	3500		3750	Kg
Goat meat	6000	6000		6000	Kg
Chicken	1500	1200		1350	Kg
**Oils/Fats**					
Palm oil	1500	1600	1400	1500	Liter
Peanut oil	1100	1125	1175	1133	Liter
Vegetable oil	1100	1200	1000	1000	Liter
**Fish and seafood**					
Sardine fish	450	400	350	400	Kg
‘Dieye’ fish	650	650	650	650	Kg
‘Guiss’ fish	800	800		800	Kg
Smoked fish	675	775	800	750	Kg
Dried fish	1200	1250	2000	1483	Kg
Sardines	2400	2400	2400	2400	Kg
Dried shrimp	5000	6000	9000	6667	kg

Pagne	2250	2400	5000	3217	Kg
Yokhoss	8600	8900		8750	Kg
Cymbium	2750	2500	3000	2750	Kg
**Sweets**					
Sugar	650	625	700	658	Kg
Chocolate	1800	1900	2000	1900	Kg
**Eggs**					
Eggs	1600	1700		1650	Kg
**Spices, condiments, and beverages**					
Pepper	3000	3850	4000	3617	Kg
Fermented néré	1000	1200	1100	1100	Kg
Salt	110	100	90	100	Kg
Garlic	1500	1300	1400	1400	Kg
Tea	100	125	150	125	Kg
Nana	225			225	Kg
Coffee	1500	1800	2000	1900	Kg
Fresh chili peppers	1500	2000	2600	2033	Kg
Dried chili peppers	3000	2900	3100	3000	Kg
Bay leaves	1700	2000	2200	1967	Kg
